# Optic disc drusen and scleral canal size – protocol for a systematic review and meta-analysis

**DOI:** 10.3389/fopht.2023.1256397

**Published:** 2023-10-05

**Authors:** Aliénor Vienne-Jumeau, Dominique Brémond-Gignac, Matthieu P. Robert

**Affiliations:** ^1^ Department of Ophthalmology, Necker-Enfants Malades University Hospital, Assistance Publique - Hôpitaux de Paris (AP-HP), Paris, France; ^2^ Institut national de la santé et de la recherche médicale (INSERM), UMRS1138, Team 17, From Physiopathology of Ocular Diseases to Clinical Development, Sorbonne Paris Cité University, Centre de Recherche des Cordeliers, Paris, France

**Keywords:** optic nerve head drusen, optic disc drusen, scleral canal, Bruch’s membrane opening, disc size, crowded disc

## Abstract

**Background:**

Around one in forty patients are diagnosed with optic disc drusen (ODD) during their lifetime. Complications of these acellular deposits range from asymptomatic visual field deficits to artery occlusion and subsequent cecity. Still, the pathogenesis of their emergence remains controversial. In particular, it was suggested 50 years ago that a narrow disc and scleral canal is one factor leading to axoplasmic flow disturbance, which induces ODD formation. However, this hypothesis is still debated today. To evaluate the basis of this theory, we will conduct a systematic review and meta-analysis of studies evaluating the scleral canal size in patients with ODD and in healthy subjects.

**Methods:**

We will search MEDLINE via PubMed, Cochrane, and EMBASE electronic databases to identify articles published before November 29, 2022 that measure the scleral canal size in patients with ODD and in healthy subjects. In addition, grey literature will be searched. The meta-analysis will include studies that include patients with a clinical or imaging diagnosis of ODD and healthy subjects. Additionally, we will perform a subgroup analysis to compare patients with buried ODD and patients with visible ODD. Extracted data from included studies will be presented descriptively, and effect sizes will be computed based on the recommendations from the Cochrane Collaboration handbook.

**Discussion:**

The hypothesis that a narrow scleral canal is a risk factor of ODD has long been debated and this systematic review and meta-analysis should disentangle the different views. Understanding the underlying factors driving the development of ODD should help us focus on patients at risk and develop strategies to prevent advanced stages of the disease in these patients. Besides, focusing on patients with small scleral canals should help us derive associated factors and provide a better understanding of the pathology.

**Systematic review registration:**

https://www.crd.york.ac.uk/prospero/display_record.php?ID=CRD42022375110.

## 1 Introduction

Optic disc drusen (ODD) are acellular deposits that are thought to result from axonal disintegration following axoplasmic flow disruption in the optic nerve head (1). The reported prevalence in adults varies from 0.2% (2) to around 2.0% (3, 4). Only 0.4% of children are thought to be affected. The diagnosis is often made incidentally in children with pseudo-papilloedema (5, 6) or in adults with visible drusen overlying the border of the disc. However, more than half of the patients have visual fields deficits (blind spot enlargement, field constriction) due to retinal nerve fiber layer atrophy (7) and a small number of patients with large ODD will develop dramatic complications, such as choroidal neovascularization, central artery occlusion or anterior ischemic neuropathy (8–12). Understanding underlying risk factors could allow clinicians to screen patients at risk and undertake a more specific follow-up to evaluate the evolution of the ODD and their consequences.

It has long been proposed that ODD are more likely to emerge in patients with a narrow scleral canal, as the latter is the location of increased axonal mechanical constraints. Several studies have been undertaken to test this hypothesis, but with diverging results (13–15). However, several factors – including the location of the ODD, the age of the patients, the instrument for measurement – are likely to influence the outcome. Therefore, the association between the presence of ODD and the size of the disc and scleral canal would be worth exploring in a systematic way.

The anterior opening of the optic nerve scleral canal is, by definition, the anatomic entrance to the scleral canal at the level of the sclera. It is mostly evaluated using either fundus pictures, where it corresponds to the limits of the disc, or optical coherence tomography (OCT). In most studies, measurements at the level of the Bruch’s membrane opening (BMO) are considered as proxies of the measurements at the level of the anterior opening of the optic nerve scleral canal (16, 17). Indeed, the BMO is well defined on OCT (14, 15, 18, 19) and seems to remain stable over time and conditions (17, 20). The high-resolution enhanced depth imaging spectral-domain OCT (EDI SD-OCT) and swept source OCT (SS-OCT), in particular, provide a greater penetration and a better characterization of deep structures, with less artefacts induced by the drusen themselves (21). EDI-SD-OCT with scan averaging is the ODD diagnostic modality recommended by the Optic Disc Drusen Studies Consortium (22). It has proven equivalent to SS-OCT in that regard (21).

This systematic review and meta-analysis will thus aim at evaluating the mean difference of the scleral canal size at the level of the BMO between patients with ODD and healthy controls, with a secondary focus on patients with buried ODD versus patients with visible ODD.

Two main objectives will be evaluated:

- Mean difference of the scleral canal size at the level of the BMO using fundus pictures between patients with ODD and healthy controls.
- Mean difference of the scleral canal size at the level of the BMO using OCT (SD-EDI or SS) between patients with ODD and healthy controls.

Because we expect that patients with buried ODD and patients with visible ODD might differ, we will also undergo a subgroup analysis and compute the following outcomes:

- Mean difference of the scleral canal size at the level of the BMO using fundus pictures between patients with buried ODD and patients with visible ODD.
- Mean difference of the scleral canal size at the level of the BMO using OCT (SD-EDI or SS) between patients with buried ODD and patients with visible ODD.

## 2 Methods/design

The literature search and analysis will follow the Preferred Reporting Items for Systematic Reviews and Meta-Analyses (PRISMA) (23) (see **Supplementary File 1**) and Meta-analysis of Observational Studies in Epidemiology (MOOSE) (24) guidelines.

### 2.1 Search strategy

We will search MEDLINE via PubMed, Cochrane, and EMBASE electronic databases to identify articles published before November 29, 2022 that measure the mean difference of the scleral canal size at the level of the BMO between patients with ODD and healthy controls or between patients with buried ODD and patients with visible ODD. In addition, grey literature will be searched in Google Scholar, Greylit.org, World Health Organization Clinical Trials Search Portal, ClinicalTrials.gov and the European Union Clinical Trials Register. All reference lists and bibliographies of included studies will be reviewed for potentially relevant studies that could be missed by this literature search.

The search will involve the following MeSH keywords: optic AND (disk OR disc OR nerve) AND drusen AND (canal OR area OR size OR measure OR crowded OR small).

### 2.2 Inclusion criteria

Randomized controlled and non-randomized controlled trials, as well as observational studies will be eligible for inclusion. Inclusion criteria will be patients with a clinical or imaging (autofluorescence, B-scan ultrasound, OCT, CT scan) diagnosis of ODD.

### 2.3 Exclusion criteria

Articles with previously published data (review, meta-analysis, follow-up study) and case reports will be excluded. We will exclude articles of studies that do not include people with ODD, that do not quantify the size of the scleral canal, that do not have a control group (either HS for patients with ODD or visible ODD for patients with buried ODD) or that include only syndromic ODD (ODD associated to a known predisposing syndrome, such as Pseudoxanthoma Elasticum, Retinitis Pigmentosa, Usher syndrome, Down Syndrome, Alagille Syndrome, Noonan syndrome).

We will exclude from the meta-analysis (but include in the systematic review and the sensitivity analysis) studies relying on time-domain OCT (TD-OCT) or non-EDI SD OCT for performing the measurements of the scleral canal size at the level of the BMO. Likewise, for the second main objective and subgroup analysis (measurements based on OCT), only the studies relying on gold standards state-of-the-art OCT (EDI SD OCT or SS OCT) to exclude ODD and define normal optic nerve according to the Copenhagen Consortium (15) will be included. Articles that do not provide appropriate data for pooling the outcomes despite authors being contacted for missing material will also be excluded. Data (reported or obtained from one of the authors) will be considered sufficient in one of the three following situations: sample sizes, means and standard deviations for both groups considered; sample sizes, medians and all four quartiles for both groups considered; raw values for every patient for both groups considered.

### 2.4 Review process

**Figure 1:**
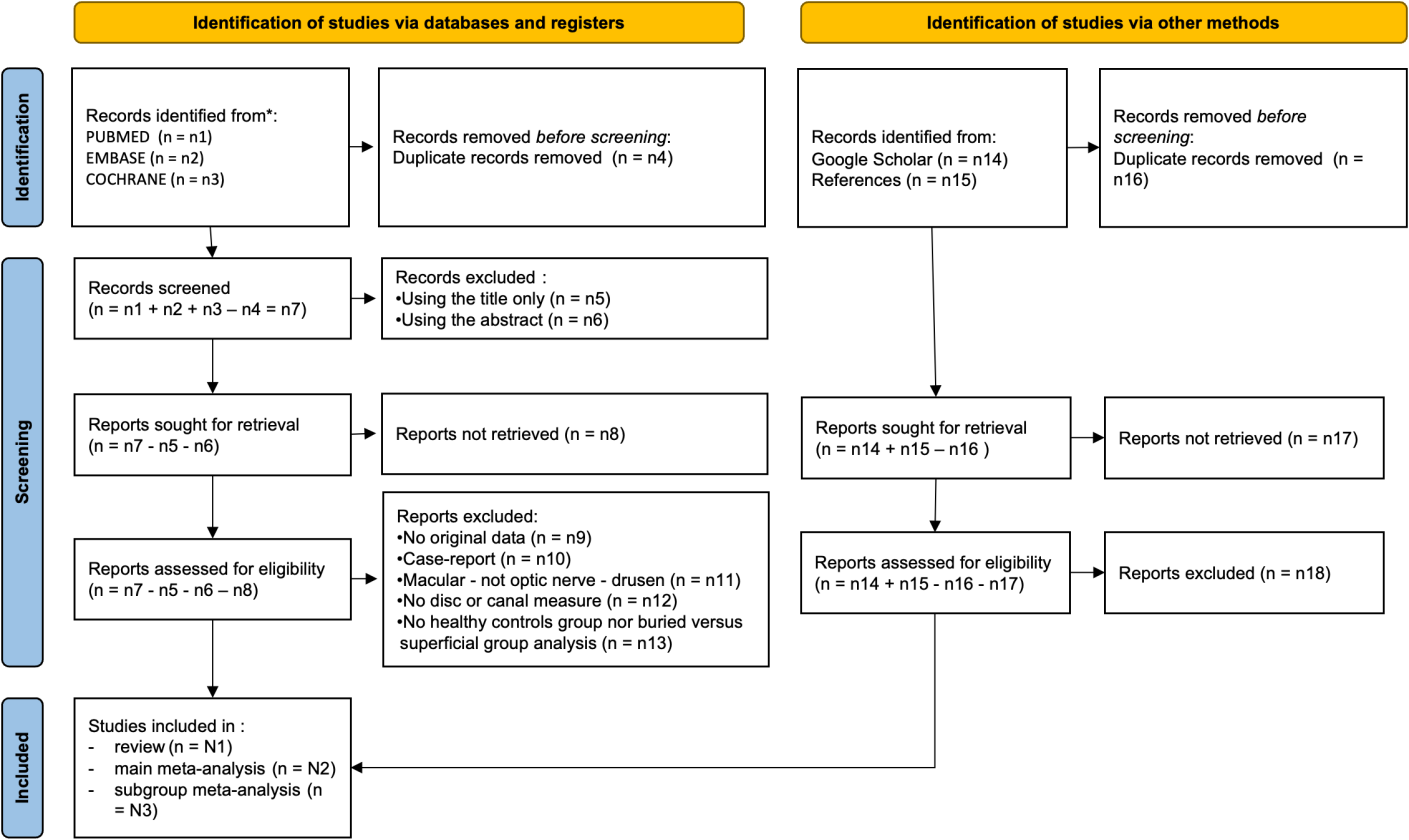
PRISMA flow chart of the review process.

**Figure 1** is a PRISMA flow chart of the review process. Potentially eligible studies will be screened for eligibility by AVJ. We will import articles to Zotero, and all articles will be reviewed (title, abstract and main text when needed) to discard those that do not meet the criteria. Data of included papers will then be extracted and the studies will be assessed for risk of bias.

### 2.5 Risk of bias appraisal

We will assess the quality of included studies through a domain-based quality assessment grid adapted from the National Institutes of Health quality assessment tool of case-control studies (25, 26). The assessment will be performed independently by two review authors (AVJ and MR), each blinded to the score given by the other. They will later discuss discrepancies until they reach consensus. If no consensus is reached, a third author (DBG) will arbitrate. Publication risk of bias will be characterized using Egger’s statistical test and visual inspection of the funnel plot, which represents the estimated effect size (horizontal axis) versus its standard error mean (vertical axis). Asymmetry of the inverted funnel shape favors publication bias.

### 2.6 Data extraction and analysis

#### 2.6.1 Study review

Upon selecting articles for inclusion, all references will be imported in Microsoft® Excel (version 16.65) for data extraction. One assessor (AVJ) will extract and collate information. Another assessor (MR) will verify the extracted material from all included articles. The following data will be extracted (see **Supplementary File 2**):

- Study characteristics: authors, title, year of publication, inclusion and exclusion criteria, sample size;
- Population characteristics: percentage of buried versus visible drusen, age, spheric equivalent
- Outcome measure characteristics: type of the parameter, means and standard deviations (or median and interquartile range (IQR)), OCT type if appropriate, magnification correction formula if applied
- Statistical analysis: test for normality, test used, whether correction for multiple comparison was applied, confounding factors, parameter significance.

The data extraction tables will be pilot-tested and refined before extraction.

The means and standard deviations will be extracted when available. If the results are reported using medians and IQR, we will search the protocol – if available – and methods to determine whether the data was shewed or whether it was a preference of the authors and there had been no test of normality although the sample size was large enough to expect a Gaussian distribution. In case the choice is not explained and the sample size is above 50, we will suppose a normal distribution and apply the following transformation formulæ: mean=median  and $SD=\frac{IQR}{1.35}$ . In any other case, we will use the formulæ by Luo et al. (27) and Shi et al. (28):


$$mean=w_{1}\left(\frac{a+b}{2}\right)+\;w_{2}\left(\frac{q_{1}+q_{3}}{2}\right)+\left(1-w_{1}-w_{2}\right).median$$

where $w_{1}=\frac{2.2}{2.2+n^{0.75}}\;\text{and }w_{2}=0.7-\frac{0.72}{n^{0.55}}.$

and $SD=\frac{b-a}{{\text{θ}}_{1}\left(n\right)}+\frac{q_{3}-q_{1}}{{\text{θ}}_{2}\left(n\right)}$

where ${\text{θ}}_{1}\left(n\right)=\left(2+0.14n^{0.6}\right).\phi^{-1}\left(\frac{n-0.375}{n+0.25}\right)$ , ${\text{θ}}_{2}\left(n\right)=\left(2+\frac{2}{0.07n^{0.6}}\right).\phi^{-1}\left(\frac{0.75n-0.125}{n+0.25}\right)$ and $\;\phi^{-1}\left(z\right)$ is the upper z^th^ percentile of the standard normal distribution, and a is the minimum value, q_1_ the first quartile, q_3_ the third quartile and b the maximum value.

If neither one of those data is available, the raw data will be sought and retrieved. If none of this material is available, it will be requested from the corresponding author (he will be contacted up to three times via e-mail). If this latter cannot provide the information, the study will be excluded from the meta-analysis.

When data are not available in the main text, we will search **Supplemental Materials** for more detailed information. If data are only available by graphical representation, the assessors (AVJ and MR) will use Plot Digitizer to extract data from graphs: the final value will be the mean of these two extractions.

#### 2.6.2 Strategy for data synthesis

Extracted data from included articles will be presented descriptively, and effect sizes will be computed based on the recommendations from the Cochrane Collaboration handbook. and Cochrane Review Manager v5.3.

Our preliminary search suggests that the mean diameter and the total area of the optic disc are two common parameters used to describe the optic disc size. Because the calculation of the mean diameter is more straightforward, we will report the mean diameter only. In cases where the mean diameter is not reported, we will transform the reported measure using the following formulas, which suppose that the optic disc can be approximated by a disc (29):

- The reported measure is the maximal and minimal diameters: $\text{mean diameter }=\frac{\text{ maximal diameter }+\text{ minimal diameter }}{2}$

- The reported measure is the horizontal diameter: mean diameter =horizontal diameter

- The reported measure is the total area: $\text{ mean diameter }=2\times \sqrt{\frac{total\;area}{\pi }}$ .

Extracted data will then be pooled to derive Hedge’s standardized mean difference. We will apply a fixed-effects model when the I^2^, the percentage of variation across studies due to heterogeneity rather than chance, was low to moderate (I^2^< 50%) (30, 31); otherwise, we will perform a random-effects model. The 95% confidence interval excluding the null value will be considered significant. The between-study variance, τ^2^, will be estimated using the Restricted Maximum-Likelihood formula (30).

We will use R v4.0.3 with the ‘Metafor’ package for the statistical analysis and the plots.

#### 2.6.3 Sensitivity analysis

Two sensitivity analysis will be performed.

First, we will explore the impact of the hypothesis that the disc can be approximated by a disc. To that end, we will analyse only studies that computed the mean diameter.

Second, we will explore the impact of choosing only studies with recent state-of-the-art OCT modalities. To do so, we will add studies using TD-OCT and non-EDI SD OCT to the analysis.

In both cases, reporting will be done in a summary table.

The overall quality of the evidence for each outcome will be evaluated by using the GRADE criteria following the Cochrane Collaboration recommendations if enough RCTs and interventional studies are included (32).

## 3 Discussion

Identifying the factors responsible for the emergence of ODD may help develop a better screening protocol and prevent dramatic complications through earlier diagnosis and care. We are not aware of any means to enlarge the scleral canal: therefore, it would not be a modifiable risk factor. Neither are we able to predict the impact of widening the scleral canal. However, should this study support the association between a narrow scleral canal and the presence of ODD, it would allow defining a better population for studies evaluating the impact of modifying other potential risk factors or introducing preventive treatments. In that regard, the potential interest of lowering the intra-ocular pressure is still pondered (33) and neuroprotective treatments are being developed, which might also prove useful to halt the progressive atrophy in patients with ODD (34–36).

Several observations support the hypothesis that a narrow scleral canal plays a central role in the formation of ODD. Genetic factors have been incriminated, which follow an irregular autosomal dominant pattern, and small optic discs have been observed in affected families (1). ODD are mainly found in caucasians, who have a smaller optic disc compared to African and Asian people (37, 38). ODD are more frequent in rod-cone dystrophies, and in particular in Usher syndrome, where scleral canals have been found smaller than in other dystrophies (39). In healthy subjects, the optic disc size correlates to the axial length (40). It is therefore interesting to note that the prevalence of ODD in nanophtalmos and posterior microphthalmos is higher than in the general population (41–43). In nanophtalmos, the presence of ODD correlates to the axial length (41). Pseudoxanthoma elasticum is another disease associated to the presence of ODD (44). If, to our knowledge, no direct link has been unveiled with the scleral canal size, it is remarkable that this pathology is characterized by ectopic mineralization in elastic fibers, and in particular in the Bruch’s membrane, which then becomes rigid. We can suppose that its opening turns out to be a zone of higher constraint for the nerve fibers.

Other hypotheses have been put forward: in particular, it has been proposed that ODD emerge from abnormal vasculature and branching, as higher frequencies of trifurcation and cilioretinal arteries have been observed in patients with ODD (45, 46). An abnormal permeability and a deficient blood barrier would induce chronic ischemia and calcium deposition, leading to ODD formation. Still, an association has been found between a small scleral canal and vascular anomalies in ODD patients (47), and it is possible that abnormal vessels are a consequence of the higher constraints induced pre- and post-natally by a narrow canal.

We acknowledge several limitations to this study. Although we will adhere to the PRISMA guidelines and methodology, it is not possible to completely account for the limitations of included studies. We expect moderate to high heterogeneity because of several variable factors, including patients’ age, measurement methods or magnification correction. However, these factors will be discussed in the narrative review, which will allow us to examine the results accordingly. A subgroup analysis taking into account the expected difference between buried ODD and visible ODD might help us explain part of the heterogeneity and the divergency observed in the literature. To limit the file drawer problem which results in publication bias, grey literature will be searched in addition to traditional databases of published literature.

## Data availability statement

The original contributions presented in the study are included in the article/**Supplementary Material**. Further inquiries can be directed to the corresponding author.

## Author contributions

AV-J: Conceptualization, Writing – original draft, Writing – review & editing. DB-G: Conceptualization, Writing – review & editing. MR: Conceptualization, Writing – review & editing.
