# Supplementary material for: Optic disc drusen and scleral canal size – protocol for a systematic review and meta-analysis
Source: Front Ophthalmol (Lausanne). 2023 Oct 5;3:1256397. doi: 10.3389/fopht.2023.1256397 (PMC11182308; doi:10.3389/fopht.2023.1256397)
Supplement: Supplementary file 2 [file Table_2.docx]

# Supplemental information 2: Standardized form for data extraction

| Theme | Data | Unavailable |
| --- | --- | --- |
| **Study characteristics** | | |
| authors |  | ☐ |
| title |  | ☐ |
| year of publication |  | ☐ |
| inclusion criteria | ☐ adults with a diagnosis of ODD | ☐ |
| exclusion criteria | previously pulished data ☐  case reports ☐  no people with ODD ☐  no quantification of the size of the scleral canal ☐  no control group ☐  only syndromic ODD ☐ | ☐ |
| sample size | ODD: (if available: buried: visible: )  HS: | ☐ |
| **Population characteristics** | | |
| participants’ gender | ODD: Male: Female:  if available:  buried: Male: Female:  visible: Male: Female:    HS: Male: Female: | ☐  ☐  ☐  ☐ |
| age | ODD:  if available:  buried:  visible:    HS: | ☐  ☐  ☐  ☐ |
| refraction error (spheric equivalent) | ODD:  if available:  buried:  visible:    HS: | ☐  ☐  ☐  ☐ |
| eye selection | ODD:  ☐ both eyes  ☐ random  ☐ right eye  ☐ the most pathological  ☐ other  HS:  ☐ both eyes  ☐ random  ☐ right eye  ☐ other |  |
| exclusion criteria | ODD:  if available:  buried:  visible:    HS: | ☐ |
| **Canal size parameter** | | |
| name | ☐ mean diameter  ☐ horizontal diameter  ☐ vertical diameter  ☐ total area | ☐ |
| method for computing |  | ☐ |
| **Sensor characteristics** | | |
| type | ☐ fundus photography  ☐ TD OCT  ☐ SD OCT  ☐ EDI SD OCT  ☐ SS OCT | ☐ |
| brand |  | ☐ |
| correction for magnification |  |  |
| **Statistical analysis** | | |
| test for normality |  | ☐ |
| tests used |  | ☐ |
| correction for multiple comparison |  | ☐ |
| confounding factors |  | ☐ |
| parameter significance |  | ☐ |
| Possible inclusion for meta-analysis | ☐ yes, without the authors’ data transmission  ☐ yes, with the authors’ data transmission  no, do not provide sufficient data ☐ | |

ODD: optic disc drusen; TD: time-domain; SD: spectral-domain; EDI: enhanced depth imaging; SS: swept-source; OCT: optical coherence tomography
